# Nowcasting Intraseasonal Recreational Fishing Harvest with Internet Search Volume

**DOI:** 10.1371/journal.pone.0137752

**Published:** 2015-09-08

**Authors:** David W. Carter, Scott Crosson, Christopher Liese

**Affiliations:** Social Science Research Group, NOAA Southeast Fisheries Science Center, Miami, Florida, United States of America; North Carolina State University, UNITED STATES

## Abstract

Estimates of recreational fishing harvest are often unavailable until after a fishing season has ended. This lag in information complicates efforts to stay within the quota. The simplest way to monitor quota within the season is to use harvest information from the previous year. This works well when fishery conditions are stable, but is inaccurate when fishery conditions are changing. We develop regression-based models to “nowcast” intraseasonal recreational fishing harvest in the presence of changing fishery conditions. Our basic model accounts for seasonality, changes in the fishing season, and important events in the fishery. Our extended model uses Google Trends data on the internet search volume relevant to the fishery of interest. We demonstrate the model with the Gulf of Mexico red snapper fishery where the recreational sector has exceeded the quota nearly every year since 2007. Our results confirm that data for the previous year works well to predict intraseasonal harvest for a year (2012) where fishery conditions are consistent with historic patterns. However, for a year (2013) of unprecedented harvest and management activity our regression model using search volume for the term “red snapper season” generates intraseasonal nowcasts that are 27% more accurate than the basic model without the internet search information and 29% more accurate than the prediction based on the previous year. Reliable nowcasts of intraseasonal harvest could make in-season (or in-year) management feasible and increase the likelihood of staying within quota. Our nowcasting approach using internet search volume might have the potential to improve quota management in other fisheries where conditions change year-to-year.

## Introduction

All federally-managed saltwater fisheries in the United States have a “hard” cap or quota on harvest and measures to prevent the quota from being exceeded. The commercial and recreational fishing sectors are held accountable for exceeding their share of the quota with adjustments in the following year [1, 2]. Commercial fishing catch statistics are typically available in time to monitor the progress towards the commercial quota share within the season. However, estimates of recreational harvest are often not available until after the season has ended. For example, the Marine Recreational Information Program (MRIP) of the U.S. National Oceanic and Atmospheric Administration (NOAA) aims to release preliminary estimates of recreational fishing harvest 45 days after the estimation period ends. Final harvest estimates are usually available by mid-April of the following year. In these situations, fishery managers seeking to determine if the quota has been exceeded can either wait for official estimates or use an alternative approach to approximate current harvest. Simple *naive* predictions can be used whereby harvest during the same period in the previous year is used to estimate the missing current period harvest level. We use the term naive from time series analysis to denote a forecast based on a previous period [3]. This practice can work well for relatively stable fisheries. However, when the season length is subject to change and fishing effort, catch rates, or fish sizes vary, the practice of using historical data to proxy the present is problematic. In addition, naive predictions for a current period are not possible when the fishery was closed during that period in the prior year.

We introduce a regression approach that uses information on fishery-related internet search volume to provide more timely intraseasonal predictions of recreational harvest. There is a growing literature showing that the internet search volume on a particular topic (e.g., unemployment insurance) can be used to predict current levels of policy-relevant variables (e.g., unemployment rates) [4]. Importantly, these predictions are not forecasts of future conditions, but rather “nowcasts” of current conditions. In the words of Choi and Varian [5] the goal is to “predict the present.” Nowcasting has value because traditional methods of compiling statistics from surveys or official records takes time. Any lag between the time that decision makers need information and the time that the information is available increases uncertainty, which increases the risk of poor decision making. If nowcasting can reduce this uncertainty, the quality of decisions can be improved [5–9]. Nowcasting attempts to arrive at an estimate sooner, but in no way replaces traditional methods, which remain the standard against which nowcasts are judged and continuously re-calibrated.

We demonstrate the harvest nowcasting approach with the Gulf of Mexico recreational red snapper fishery. This fishery has been particularly difficult to manage with progressively shortening seasons in the presence of changes in effort and an increasing average fish size. The recreational sector has overharvested red snapper in every year from 2007 to 2013 with the exception of 2010, when the Deepwater Horizon (DWH) oil spill forced a mandatory closure of prime fishing grounds during the busy summer season.

NOAA fisheries forecasts recreational harvest of red snapper and other key species several months in advance in order to set fishing seasons [10]. Forecasts are based on trends in historical catch rates and fish weight by fishing sector (private, for-hire). The agency does not regularly monitor the harvest of red snapper within the season because the season is shorter than the data reporting period. However, there have been times when managers reopened the fishery later in the year. In these cases there was a need for information on the cumulative level of harvest before the harvest data were available. For example, in 2013 following a new stock assessment, the recreational quota increased after the summer season closed and there was an interest in re-opening the season in the fall. In this case, there was a need to determine whether the new quota had been exceeded during the original season. Our proposed approach is designed to warn fishery managers of pending quota overages within the year by nowcasting harvest using a regression model including data on internet searches during the summer months when fishing activity peaks.

## Materials and Methods

### Recreational Fishery Harvest Data

Our recreational fishing harvest data series comes from the Annual Catch Limit (ACL) database assembled by the NOAA Southeast Fisheries Science Center. This database is used to monitor recreational harvest as the season proceeds in two-month waves and is compiled from three separate surveys. The NOAA MRIP provides bi-monthly (wave) estimates of fish harvested by for-hire charter and private boats fishing in the marine waters of Florida, Alabama, Mississippi, and Louisiana. Preliminary wave estimates from the MRIP are typically available 45-days after the end of the wave and final estimates for the previous year are usually released during April. Harvest estimates from private and charter boats fishing in the marine waters of Texas are provided by the Texas Parks and Wildlife Division (TPWD) Creel Survey. The TPWD estimates can be delayed anywhere from six months to a year or more. The NOAA Southeast Headboat Survey (HBS) provides estimates of harvest from head boats in the marine waters of all states in the Gulf of Mexico. The HBS estimates are also often delayed by more than a year.

Our analysis uses bi-monthly (wave) estimates of aggregate recreational fishing harvest (whole weight) of red snapper in the Gulf of Mexico between 2004 and 2013. In 2013, MRIP implemented an improved sampling design for the onsite Access Point Angler Intercept Survey (APAIS) that is used to estimate the mean harvest per angler fishing trip. Revised harvest estimates for 2013 were released in December of 2014 along with re-calibrated estimates of harvest for 2004 to 2012. However, the nowcasting thought experiment used to test competing forecast models used in this analysis requires that we develop models using only the information available at the time of the forecast.

**Fig 1:**
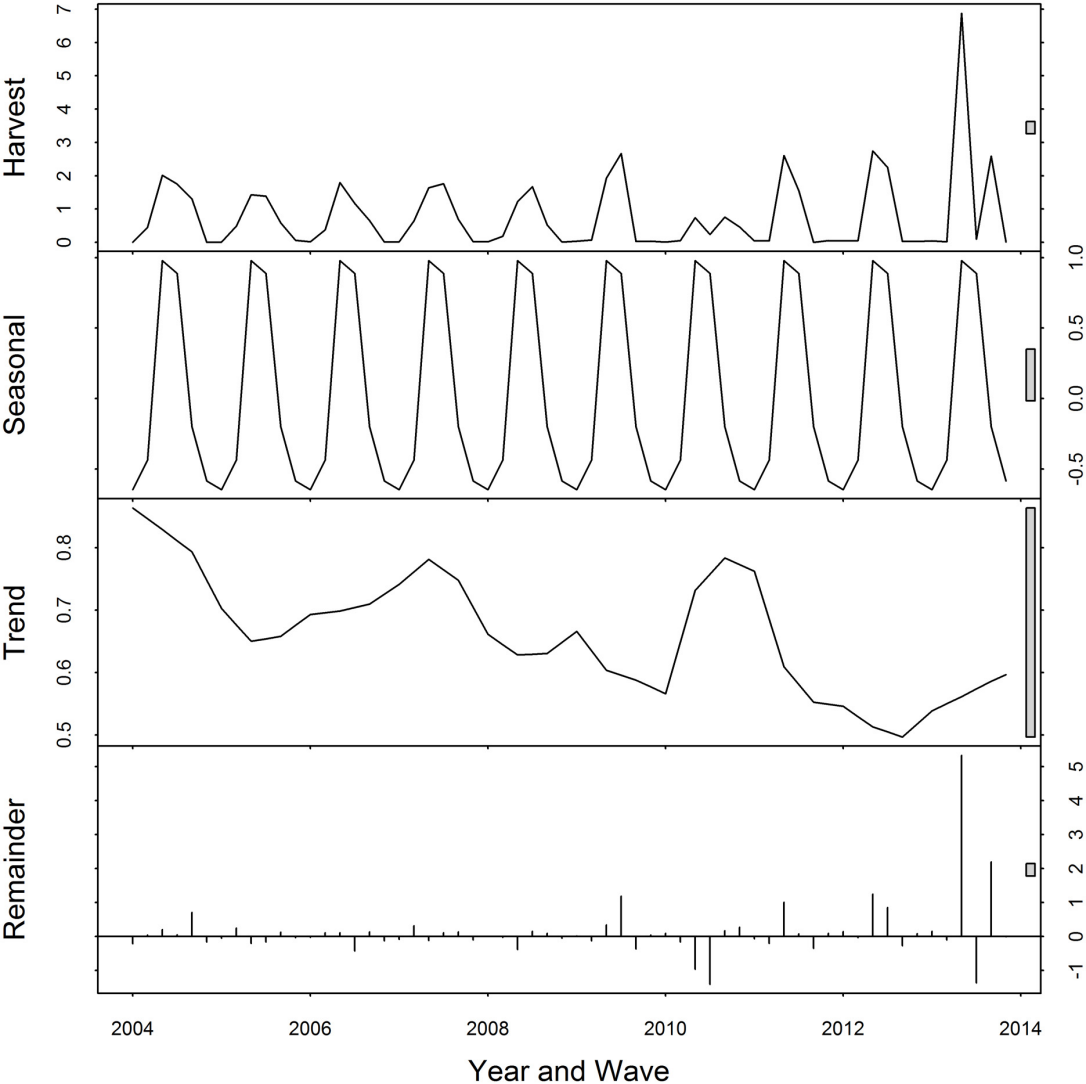
Loess Decomposition of Red Snapper Harvest (Million Pounds): 2004—2013, by wave.

The seasonal and trend Loess-decomposition of the harvest series is displayed in Fig 1. The decomposition was conducted using the stl() function in the stats package of **R** [11]. The raw harvest data series is shown in the first panel with the grey range bar on the right side indicating the relative scale of the series. The grey range bars on the right in the bottom three panels indicate the scale of each time series component relative to the raw data series with shorter range bars indicating a greater contribution. According to the second panel, red snapper harvest is highly seasonal with a peak occurring during the summer months and low during the winter months. The third panel shows what appears to be a downward trend. However, the relatively long range bar, i.e., small magnitude, suggests that the trend is not a very important contributor to the variation of the harvest series. The last panel displays the remaining random component of the series that cannot be explained by seasonality or a trend. The short range bar of the remaining component suggest that there is still considerable unexplained (beyond seasonal and trend) variation in the harvest series, especially in recent years when the fishing season has changed frequently. Some of this extra variation is evident in the reduced harvest levels during the summer of 2010 when a substantial portion of the Gulf of Mexico was closed to fishing following the explosion of the DWH oil rig.

The historic red snapper fishing seasons and bag limits for the federal waters of the Gulf of Mexico are shown in Table 1. The number of days that anglers can harvest red snapper in federal waters has decreased dramatically since 2007. There are also three recent years when the fishing season for red snapper was extended or re-opened in the fall. The first occurred for some weekends (Friday through Sunday) in the fall of 2010 to compensate for the lost fishing opportunities following the DWH event. The quota was not exceeded in 2010 even with the extra fall season. In 2012 the season was extended for seven days because of bad weather. The decision to extend the season was not made based on harvest data because estimates of harvest during the season were not available at the time. Rather, weather and wave data near the typical fishing grounds was used to determine the number of days during the season that were unfavorable for fishing. Ultimately, the quota was exceeded by more than one million pounds in 2012. A second fall opening happened in 2013 because fishery managers were not aware (due to the lag in harvest data reporting) that the quota had already been exceeded during the summer season. The following excerpt from a NOAA Southeast Regional Office (SERO) document illustrates the complexities inherent in the decision to add a supplemental season during the year [12]:

Previous projections estimated landings per day (in numbers) during summer when tourism is high and weather tends to be better than fall. Because the Gulf Council is proposing to reopen in September or October, landings per day are not expected to be as high as summer due to lower tourism, more severe weather, and children returning to school. Because the season has not been open in fall since 2010, and prior to then not since 2007, it is difficult to predict fall landings per day…Season lengths presented herein are contingent on previous projections accurately estimating the length of the fishing season under a 4.145 mp quota. Given inconsistent regulations and historical overages of the quota, there is potential that the summer season length (Jun 1-Jun 28) was too long and may result in a quota overage. Similarly, given the short duration of the season, it may have been too short and the quota might not have been met. If landings during summer are greater than the current 4.145 mp quota, then season lengths presented herein will be overestimated. If landings during summer are less than the current 4.145 mp quota, then average weights and/or landings per day have been overestimated, and season lengths presented herein would be underestimated.

**Table 1:** Regulations on the Recreational Harvest of Red Snapper in the Federal Waters of the Gulf of Mexico, 2004—2013.

Year	Season Start	Season End	Bag Limit	Days Open
2004	04-21	10-31	4	190
2005	04-21	10-31	4	190
2006	04-21	10-31	4	190
2007	04-26	10-31	2	185
2008	06-01	08-05	2	64
2009	06-01	08-15	2	74
2010	06-01	07-24	2	52
	10-01	11-21 (Fri,Sat,Sun only)	2	24
2011	06-01	07-19	2	47
2012	06-01	07-17	2	45
2013	06-01	06-29	2	28
	10-01	10-14	2	14

Note that the process to reopen a season is a lengthy administrative process. Thus, the harvest estimates for the May-Jun wave of 2013 were not available when the decision to re-open the fall season was made. It was assumed that the quota was harvested exactly during the summer season. Data from 2011 and 2012 were used to project the harvest information necessary to set the fall season.

Though not shown in Table 1, Texas, Louisiana and Florida set regulations for fishing in state waters in some years that differed from the federal waters regulations. Throughout the study period, Texas allowed harvest year-round in state waters with a 4-fish bag limit. In 2013, Louisiana had a weekend-only (Friday through Sunday and some holidays) season in state waters from mid-April through the end of September with a 3-fish bag limit (2-fish during the federal season). Also, in 2013, Florida opened red snapper fishing in state waters for 68-days from June 1st to July 14th and from October 1st to October 14th.

### Internet Search Volume Data

Google provides two tools to examine the periodic volume of queries that users enter into the Google internet search engine. The first, Google Trends, allows users to download monthly or weekly indices of the web search volume for a particular term. The index value for each period is calculated by dividing by the total count for all queries in that period from the U.S. and then scaled such that the highest volume in the series is assigned 100 and the lowest volume is assigned 0. Importantly, the normalization controls for the growth in all Internet search use over time.

The second Google tool, Google Correlate, returns the search volume for any term along with the volume of searches for the top correlated terms. The tool will also return the top internet search terms that is correlated with any data series you enter. All series from the Google Correlate tool are further standardized to have a mean value of zero and a variance of one [13].

We downloaded the standardized monthly series from 2004 to 2013 for the term “red snapper season” in the U.S. from the Google Correlate tool. This series contains more information than the Google Trends series because Google Trends sets the search volume to 0 for periods where the volume is below a certain (unspecified) threshold. In this case, the index for the term “red snapper season” was 0 in most months prior to 2011 in the Google Trends series, but not the Google Correlate series.

There are many other search terms that might be correlated with red snapper harvest. Some are too general (e.g., red snapper) because the terms refer to behavior not related to fishing (e.g., recipes). Other terms (e.g., red snapper bag limit) are too specific because there simply is not enough search volume. We started with the most obvious term, “red snapper season”, and it worked reasonably well. As noted, above, the Google Correlate tool will return the top internet search terms correlated with any data series you enter. Table 2 shows the search terms that are correlated with the monthly (interpolated bi-monthly) red snapper harvest series. On correlation alone, this table suggests we should have used the term “coleslaw recipes” in our model. However, this term and the others in the table are not directly related to fishing. Most appear related to summer activities, suggesting a common seasonality with the red snapper harvest series. The raw correlation between the red snapper harvest series and the (1st month) “red snapper season” search term is 0.482. This correlation is much lower than the correlation with searches for “coleslaw recipes’, but the relationship is much more plausible. Since there are millions of possible search terms, correlation alone cannot identify an appropriate search term. Instead, a search term must be found that a) is fairly exclusive to the particular fishery and management problem being studied and b) is broad enough to produce significant volume. The latter implies that the particular fishery management problem being studied needs to lead anglers to query the internet. A search term satisfying both a) and b) will not necessarily exist for all fisheries and management problems.

**Table 2:** Search Terms Correlated with Red Snapper Harvest.

Correlation	Search Term
0.8006	coleslaw recipes
0.7932	stray kittens
0.7918	six flags over ga
0.7880	nebraska softball
0.7857	behr deck stain
0.7732	steel cooler
0.7731	kill poison ivy
0.7695	poisonous snakes
0.7635	sand filter
0.7612	king island
0.7607	red spider
0.7547	injured bird
0.7541	sandusky weather
0.7539	deck coating
0.7539	delonghi pinguino
0.7533	deadhead
0.7532	citronella
0.7529	cookout ideas
0.7519	ice chest
0.7510	hayward super pump

**Fig 2:**
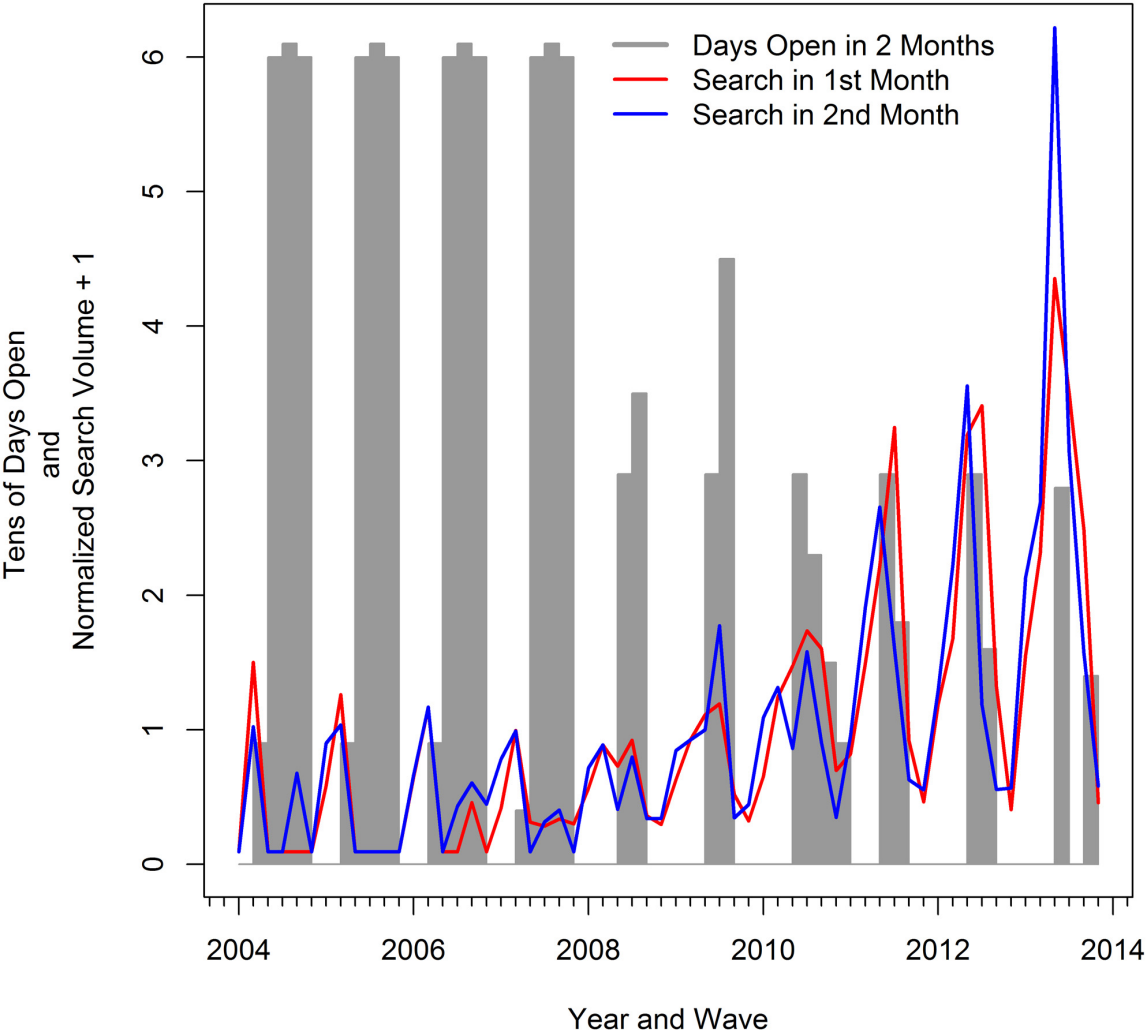
Red Snapper Season and Internet Search for Red Snapper Season: 2004—2013, by wave.

Our harvest data is in bi-monthly periods and the internet search volume data is monthly. Rather than aggregating the monthly internet search volume data to bi-monthly observations, we chose to consider the search volume of both months in a two-month period separately in our discussion and models. Fig 2 shows the U.S. search volume for the term “red snapper season” on a bi-monthly scale. The line labeled “Search in 1st Month” is the volume in the first month of the period and the line labeled “Search in 2nd Month” is the volume for the second month of the period. Note that we have added 1 to every observation in the two search volume series so that the series are everywhere positive. This is only for display purposes. In general, the search volume of the two months in each period move together. The figure also shows the number of days in each two-month period when red snapper harvest was allowed in the federal waters of the Gulf of Mexico. Notice how the searches peak just before the season opens in early years, but become more erratic in later years.

**Fig 3:**
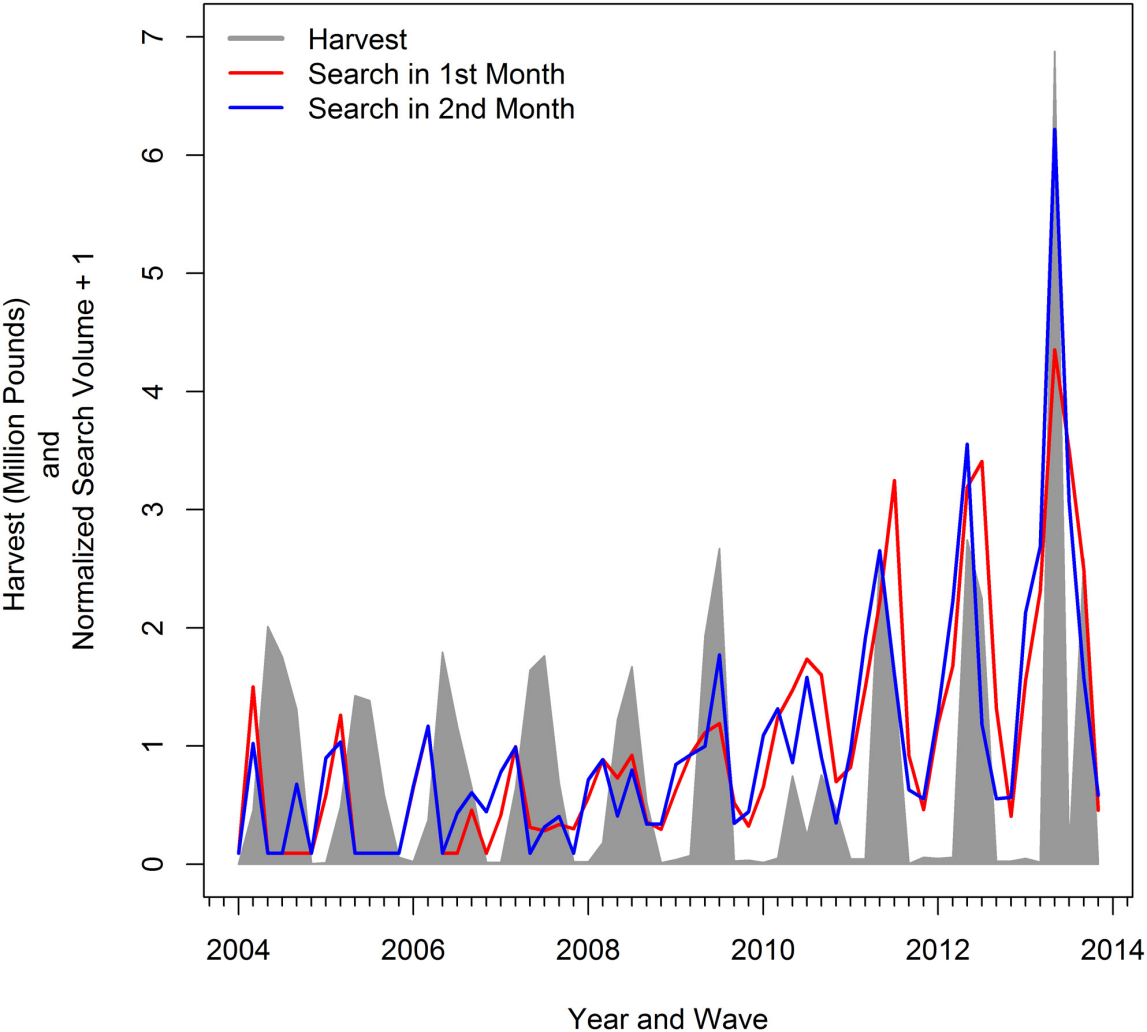
Red Snapper Harvest and Internet Search for Red Snapper Season: 2004—2013, by wave.

Fig 3 again shows the U.S. search volume for the term “red snapper season” for each month in the two-month period, this time with the bi-monthly harvest of red snapper in the Gulf of of Mexico. The search volume precedes the harvest levels in the early years of the series, but tends to overlap with harvest levels in latter years.

### Modeling Approach and Diagnostics

There is approximately a two month lag in the preliminary estimates of the recreational harvest of red snapper in the Gulf of Mexico; that is, the earliest estimated harvest for the two-month period just completed is available at the end of the following two-month period. Therefore, managers do not know whether the quota has been exceeded at the end of a period until two months later. We consider three different approaches to nowcast the harvest level of the period just completed. The approaches are based on three different forecasting models. The first model is a naive prediction that assumes the current period harvest is the same as the harvest level in the same period of the previous year. This nowcasting approach typically works well for series with stable seasonality [3] and will be the benchmark with which we evaluate the other two nowcasting models. The benchmark is set to reflect a strategy that is intuitively appealing and easy to implement. We emphasize, however, that the naive approach does not represent current practice because NOAA fisheries does not usually monitor the red snapper quota within the year.

Our next model addresses the seasonal pattern evident in the harvest time series (see Fig 1) and key fishery closure events. The seasonal pattern appears to be stable (i.e., deterministic), however, there is still a possibility that there are changes in the seasonality over time that are not revealed upon visual inspection of the series. Canova and Hansen developed a procedure to test the null hypothesis of deterministic (six two-month periods annually) seasonality against the alternative of seasonal non-stationarity [14]. The nsdiffs function in the forecast package for **R** uses this test to determine the number of seasonal differences (i.e., current period harvest minus the harvest six periods or one year prior), if any, necessary to make a given seasonal time series stationary. In the case of the red snapper harvest series, the test does not reject the null hypothesis of deterministic seasonality. Therefore, the starting point for the second model is a regression of the harvest series on an intercept and one indicator for each two-month wave (2 through 6) that equals one if the observation is in that wave and zero otherwise. We also add the first and sixth lag of the harvest variable to this regression to address seasonal autocorrelation, a variable for the number of days closed to red snapper fishing in the period, and an indicator for the two periods following the DWH oil spill event in 2010. This model nowcasts the harvest level in the current period to be the same as the historic average harvest level in that period with adjustments for the harvest levels in the previous period and a year ago as well as closures due to regulations and the DWH event. The third model adds two variables to the second model indicating the internet search volume for the term “red snapper season” in the first and second month of the period. The third model also includes interactions between the number of days closed and the search terms to allow for a differential effect of search when the season is closed and when it is not. Recent observations related to the use of internet search volume in forecasting, for example flu outbreaks, has suggested that the distinction between simple interest in a topic and actual behavior is important [15, 16]. The general form of the complete model is

$$\begin{array}{cc} h_{t}=\alpha_{1} & +\sum_{i=2}^{6}\alpha_{i}w_{it}+\beta_{1}h_{t-1}+\beta_{2}h_{t-6}+\beta_{3}d_{t}+\beta_{4}c_{t}\\ & +\beta_{5}{\text{s1}}_{t}+\beta_{6}{\text{s2}}_{t}+\beta_{45}c_{t}{\text{s1}}_{t}+\beta_{46}c_{t}{\text{s2}}_{t}+e_{t} \end{array}$$ (1)

where *h*_*t*_ is the harvest of red snapper in period *t*, each *w*_*it*_ term equals one if period *t* is wave *i* of the year and zero otherwise, *h*_*t*−1_ and *h*_*t*−6_ is the harvest of red snapper in the previous period and the same period of the previous year, respectively, *d*_*t*_ equals one if the period was closed to fishing following the DWH event and zero otherwise, *c*_*t*_ is the number of days closed to red snapper fishing during the period, *s*1_*t*_ and *s*2_*t*_ is the internet search volume for the term “red snapper season” during the first and second months of the period, respectively, *e*_*t*_ is an i.i.d. error term, and the *α* and *β* terms are parameters to estimate. To recap, the first model is a naive prediction based on the harvest during the same period in the previous year; the second model contains the wave indicator variables (*w*_*it*_), the first (*h*_*t*−1_) and sixth (*h*_*t*−6_) lags of harvest, the indicator (*d*_*t*_) for the DWH event and the variable (*c*_*t*_) for the number of days closed to red snapper fishing; and the third model is the complete equation shown above.

We evaluate the fit of each of the three model specifications and the suitability for forecasting using data from 2004 through 2011. The data for 2012 and 2013 are used later to evaluate the ability of the best fitting specification to nowcast the harvest in each two-month period of 2012 and 2013 with days open to red snapper fishing. Model specification goodness-of-fit is examined using the adjusted *R* ^2^ measure of the coefficient of determination. The residuals of a good forecasting model will have a mean of zero and be uncorrelated. If, in addition, the residuals are normally distributed and have a constant variance, then the construction of confidence intervals for forecasts is straightforward [3]. The null of independence and normality of the residuals from each forecast model are evaluated using the Ljung-Box and Shapiro-Wilk tests, respectively (box.test() and shapiro.test() in the stats **R** package). The Breusch-Pagan test (bptest() in the lmtest **R** package) is used to test the null that the variance of the residuals is constant. Unless otherwise noted, all modeling, diagnostics, and forecasting is conducted using the forecast package for **R** [17].

The performance of each model in nowcasting harvest is examined by comparing the actual harvest in periods of 2012 and 2013 that have some days open for red snapper fishing with the model predictions for these periods. For any given model the harvest for each period is predicted by estimating the model with data up to the period with open days and then calculating a one-step-ahead forecast. For example, the harvest nowcasts for May-Jun of 2012 is based on the models estimated using data up to Mar-Apr of 2012. Note that in this example the third (complete) model would use the internet search volume information for May-Jun to help predict May-Jun harvest. We compare the nowcasting ability of the second and third models to evaluate the potential difference in nowcasts with and without the internet search volume information. This follows the approach used by others who have examined whether search volume can improve the best forecasts available using the data available at the time of forecast [5, 7, 9]. Nowcast accuracy is measured using the mean absolute error (MAE) as recommended by Hyndman and Koehler [18] when comparing the accuracy of forecasts of series measured on the same scale. Lower MAE values indicate more accurate harvest nowcasts.

As a check on the policy relevance of the modeling results we examine the ability of a model to determine whether the harvest has exceeded the quota during a period with open days. Two performance measures are examined. For each model, the first performance measure takes the cumulative harvest from the previous periods and adds the one-step-ahead forecast for the current period, i.e.,

$$\hat{H_{t}}=H_{t-1}+\hat{h_{t}}$$ (2)

where $\hat{H_{t}}$ is the estimated cumulative harvest in period *t* and *H*_*t*−1_ is the observed cumulative harvest in the previous period, and $\hat{h_{t}}$ is the nowcast harvest estimate for period *t*. For each model, we graphically examine how close the estimated cumulative harvest from Eq 2 is to the quota during the open season. The nowcast estimates are subject to prediction error. Therefore, assuming that the prediction errors are normally distributed, the second performance measure for each model calculates the prediction intervals and the probability that the quota has been exceeded for each nowcast period.

## Results

### Specification Evaluation and Parameter Discussion

The parameter estimates and diagnostics for the three potential forecasting models using the data through 2011 are shown in Table 3. The first model labeled “Naive” in each table does not have any coefficient estimates because the approach simply assumes that the current wave harvest level is equal to the level in the same wave of the previous year. Estimates for the second model are shown in the column labeled “Without Search”. The last column labeled “With Search” shows the estimates for the complete model from Eq 1. The adjusted *R* ^2^ measure of fit increases with each model going from left to right. The naive model explains about 58% of the variation in red snapper harvest. The amount of variation explained increases to around 83% with the second model when the seasonality and fishery closures are included. Importantly, the complete model with the internet search volume explains about 92% of harvest variation. This suggests that the internet search volume for the term “red snapper season” can be used to predict red snapper harvest variation beyond the amount explained by seasonality and fishery closures.

**Table 3:** Red Snapper Recreational Harvest Regression Results: 2004—2011.

	Naive	Without Search	With Search
Intercept	0.000 (0.000)	0.454 (0.241)	1.354*** (0.277)
Mar-Apr		0.315 (0.168)	0.185 (0.156)
May-June		1.815*** (0.276)	1.157*** (0.289)
Jul-Aug		1.799*** (0.355)	0.919* (0.343)
Sep-Oct		0.621* (0.277)	0.119 (0.227)
Nov-Dec		0.129 (0.172)	0.048 (0.131)
Harvest(t-1)		-0.098 (0.127)	-0.021 (0.092)
Harvest(t-6)		-0.212 (0.165)	0.057 (0.148)
Deepwater Horizon		-0.990** (0.318)	-1.360*** (0.257)
Days Closed in Wave		-0.007 (0.004)	-0.023*** (0.005)
Search in 1st Month in Wave			0.148 (0.434)
Search in 2nd Month in Wave			0.890** (0.310)
Search in 1st Month x Days Closed			-0.001 (0.010)
Search in 2nd Month x Days Closed			-0.018* (0.008)
R^2^	0.581	0.863	0.942
Adj. R^2^	0.581	0.830	0.920
Num. obs.	48	48	48
RMSE	0.778	0.321	0.220
Residual mean	-0.029	0.000	-0.000
Box-Ljung p-value	0.000	0.418	0.508
Shapiro-Wilk p-value	0.000	0.000	0.006
Breusch-Pagan p-value	0.000	0.473	0.190

***p < 0.001,

**p < 0.01,

*p < 0.05.

The coefficients are scaled to million pounds.

The residual mean in all models, except the naive, is zero indicating that the models can produce unbiased forecasts. The null of independence in the Ljung-Box test cannot be rejected at the 95% level in all models, except the naive. The null of normality in the Shapiro-Wilk test is rejected at the 95% level in all models. The null of a constant residual variance (homoscedasticity) in the Breusch-Pagan test cannot be rejected at the 95% in any model, except the naive. Thus, the complete model comes close to satisfying all of the tests for a good forecast model, passing the tests for constant residual variance and residual independence, but not the test for residual normality. The normality of the residuals is not essential, but does make calculating confidence intervals easier. In any case, the complete model specification including the seasonality, the fishery closures, and the internet search volume appears to fit the historic data best and provides the best forecasting properties. Based on this finding we proceed with the complete model for the one-step-ahead nowcast of the harvest in each two-month period of 2012 and 2013 with days open to red snapper fishing. However, we also generate the nowcasts using the specification (model 2) without the internet search volume so that we can compare the nowcasts with and without the internet search volume.

Before discussing the results of the nowcasts for 2012 and 2013, we briefly review the estimated parameters from the complete specification (the last column) in Table 3. The constant and the indicators for May-Jun and Jul-Aug are significantly different from zero at the 95% level. Approximately one million pounds more of red snapper was harvested during waves 3 and 4 than during Jan-Feb. This happens because the season for red snapper occurred primarily during waves 3 and 4 in each year of our study period. Harvest decreased by more than 1.3 million pounds in the waves following the DWH event. The parameters on the harvest one wave and one year prior are not significant at the at the 95% level.

The effects of the number of days closed to red snapper fishing in a wave and the internet search volume are more complicated because of the interaction terms. At the average level of internet search volume in a period (i.e., *s*1_*t*_ = *s*2_*t*_ = 0 because the search series is normalized to the mean of the series) each day closed to fishing is related to a 23 thousand pound reduction in harvest on average. When there are no days closed to fishing in a wave then the effect of internet search volume is given by parameters on 1st and 2nd month activity alone. The parameters related to the search volume in 1st month of the wave are not significant at the 95% level. The parameters related to the 2nd month search volume are significant at the 95% level suggesting a one unit (standard deviation) increase in search volume is related to nearly 900 thousand more pounds being harvested (when every day in the wave is open to harvest). Based on the interaction terms, for each additional day open to harvest, a one unit increase in internet search volume in the 2nd month of the wave is related to a 18 thousand pound increase in harvest.

### Nowcasts for 2012 and 2013

Table 4 shows the parameter estimates for the models used to calculate the one-step-ahead forecasts (nowcasts) for the waves with open fishing days in 2012 and 2013. Each column has the results for the model estimated using the data up to the year and wave indicated. For example, the column labeled 2012.2 contains the parameter estimates based on the data through Mar-Apr of 2012. The estimates in this column are used to calculate the nowcast for May-Jun of 2012. The parameter estimates are similar to the results described for the complete model (last column) in Table 3. However, the estimates of the parameters on internet search volume terms for the 2nd month in the wave are larger for the model using the most recent data. The magnitude of the parameter on search in 2nd month in the wave and parameter on the related interaction with days closed doubles for the data through 2013.4 relative to the rest of the periods. This suggests a growing importance of the internet search volume signal. The model fit also declines slightly in recent periods indicating an increasing amount of noise in the harvest data.

**Table 4:** Red Snapper Recreational Harvest Rolling Regression Results.

	2012.2	2012.3	2013.2	2013.4
Intercept	1.355*** (0.262)	1.295*** (0.253)	1.366*** (0.238)	1.680*** (0.385)
Mar-Apr	0.180 (0.139)	0.229 (0.128)	0.129 (0.115)	0.047 (0.193)
May-June	1.152*** (0.273)	1.284*** (0.232)	1.026*** (0.212)	0.932* (0.359)
Jul-Aug	0.913** (0.326)	1.075*** (0.274)	0.829** (0.260)	0.828* (0.405)
Sep-Oct	0.115 (0.215)	0.186 (0.201)	-0.010 (0.187)	0.127 (0.285)
Nov-Dec	0.047 (0.123)	0.038 (0.122)	0.015 (0.115)	0.132 (0.195)
Harvest(t-1)	-0.021 (0.089)	-0.031 (0.088)	0.001 (0.086)	-0.157 (0.106)
Harvest(t-6)	0.059 (0.141)	-0.031 (0.102)	0.078 (0.095)	0.177 (0.153)
Deepwater Horizon	-1.362*** (0.249)	-1.233***(0.205)	-1.377*** (0.201)	-1.639*** (0.338)
Days Closed in Wave	-0.023*** (0.005)	-0.022*** (0.005)	-0.022*** (0.004)	-0.027*** (0.007)
Search in 1st Month in Wave	0.144 (0.418)	0.025 (0.397)	0.223 (0.370)	-0.102 (0.617)
Search in 2nd Month in Wave	0.892** (0.298)	0.912** (0.297)	0.733* (0.286)	1.531** (0.460)
Search in 1st Month x Days Closed	-0.000 (0.009)	0.001 (0.009)	0.001 (0.008)	0.007 (0.014)
Search in 2nd Month x Days Closed	-0.018* (0.007)	-0.018* (0.007)	-0.017* (0.007)	-0.030** (0.011)
R^2^	0.944	0.950	0.947	0.920
Adj. R^2^	0.923	0.932	0.931	0.896
Num. obs.	50	51	56	58
RMSE	0.214	0.213	0.218	0.372

***p < 0.001,

**p < 0.01,

*p < 0.05.

The coefficients are scaled to million pounds.

**Fig 4:**
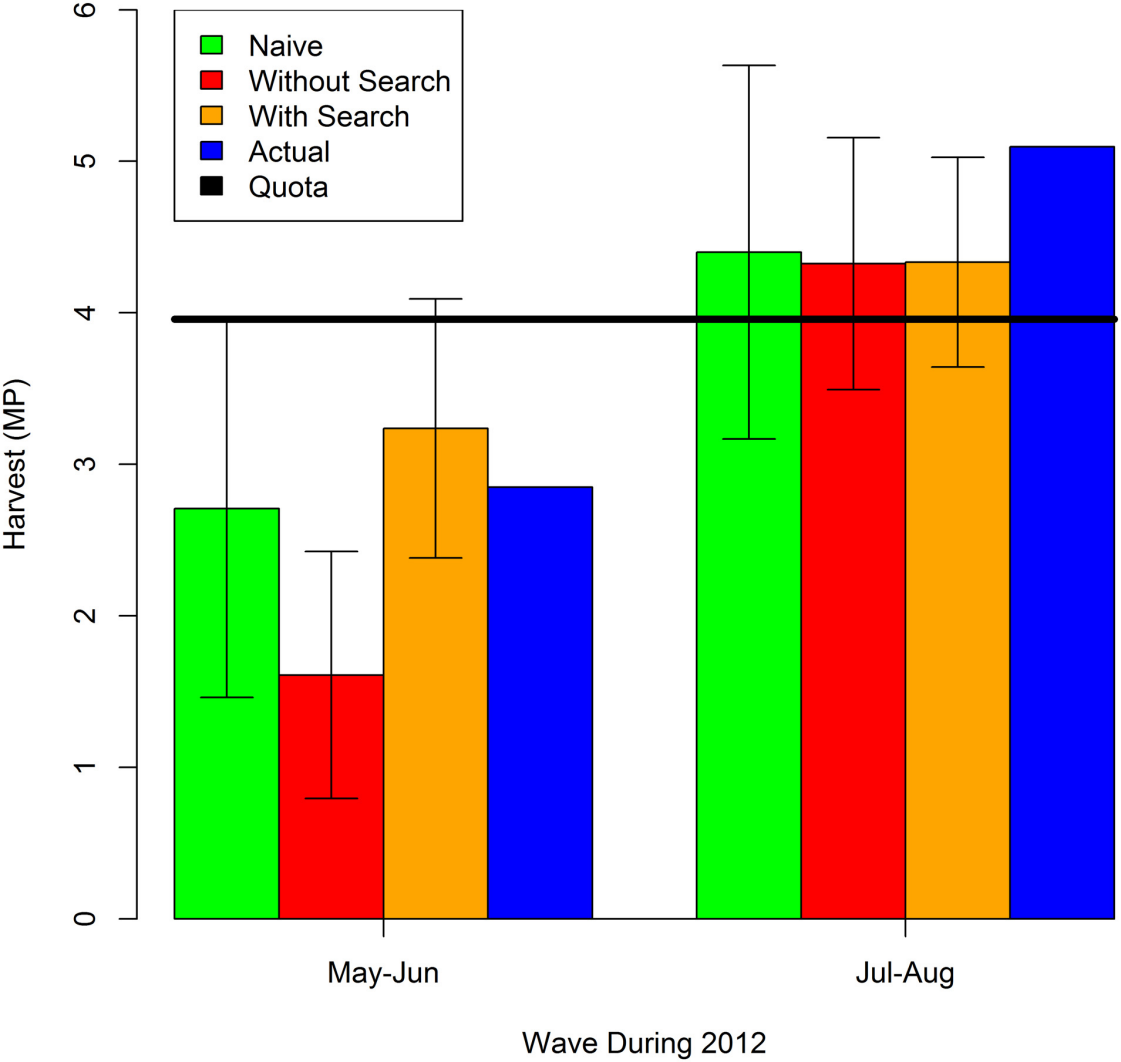
Nowcasts vs. Actual Cumulative Harvest for May-Jun and Jul-Aug of 2012.

We use Eq 2 and models 2012.2 and 2012.3 to nowcast the cumulative harvest in waves 3 and 4 of 2012. We focus on waves 3 and 4 because the harvest of red snapper in federal waters was closed in the other waves of 2012. The results are shown in Fig 4 along with the actual cumulative harvest. The 95% confidence intervals are also indicated for each nowcast. The black horizontal bar indicates the quota for the fishing year. The naive model slightly underpredicts the cumulative harvest in May-Jun while the complete model with internet search volume overpredicts. The model without internet search volume considerably underpredicts the actual harvest in May-Jun. For May-Jun of 2012, the complete model nowcasts only a 4% probability that the quota is being exceeded (in May-Jun). The nowcasts of cumulative harvest in Jul-Aug are similar across the three models and all are less than the actual cumulative harvest. For Jul-Aug of 2012, the complete model nowcasts a 86% probability that the quota is being exceeded (in Jul-Aug). The MAE of the nowcast for waves 3 and 4 of 2012 with the naive approach, the model without search volume, and the model with search volume is 0.42, 1.01, and 0.58, respectively. In the case of 2012 the naive approach nowcasts May-Jun and Jul-Aug harvest slightly better than the complete model with internet search volume. However, the model nowcasts these waves 43% better with internet search volume than without.

**Fig 5:**
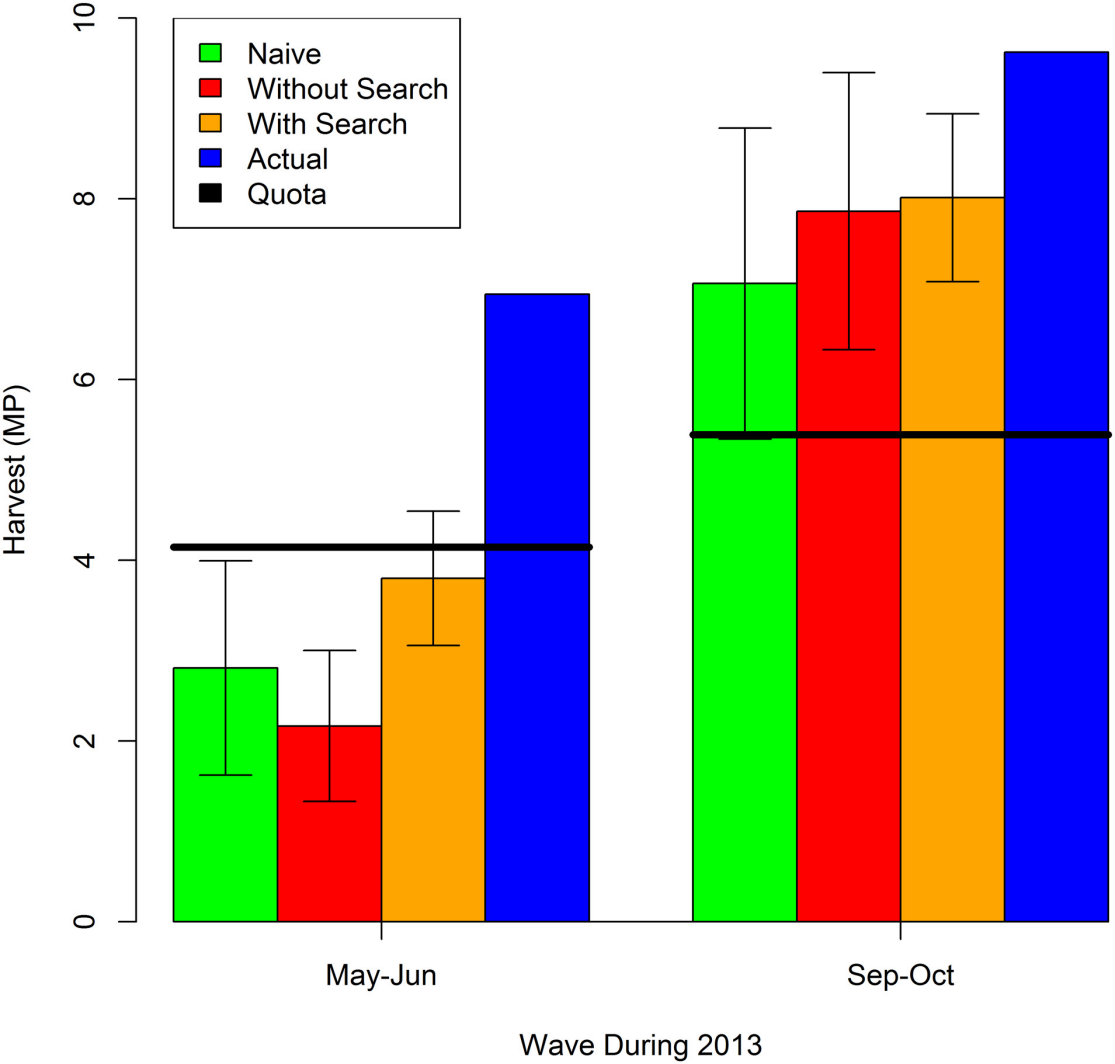
Nowcasts vs. Actual Cumulative Harvest for May-Jun and Sep-Oct of 2013.

The cumulative harvest in waves 3 and 5 of 2013 is nowcast using Eq 2 and models 2013.2 and 2013.4. The results are shown in Fig 5 along with the actual cumulative harvest, the estimate using the naive model, and the estimate using the model without the internet search volume. Again, the black horizontal bars indicate the quota which was increased once during 2013. We focus on waves 3 and 5 because the harvest of red snapper in federal waters was closed in the other waves of 2013. The 95% confidence intervals are also indicated for each nowcast. All models significantly underpredict the unprecedented cumulative harvest in May-Jun of 2013. The complete model with internet search volume comes closest and suggests a 17% probability of exceeding the quota in May-Jun of 2013. Note that due to the lag in recreational reporting, managers were unaware that the quota had been greatly exceeded in May-Jun, and reopened a second shorter season in Sep-Oct (along with a slight and unrelated increase in the quota). The nowcasts of cumulative harvest in Sep-Oct of 2013 are all less than the actual cumulative harvest, but all models suggest that the quota is being exceeded in this wave with near certainty. However, the naive approach is problematic in this case because there was no harvest in the previous year as Sep-Oct in 2012 was closed to fishing in federal waters. There was only a small amount of harvest in state waters during Sep-Oct of 2012 to be added to the actual May-Jun harvest to predict the Sep-Oct cumulative harvest for 2013. The MAE of the nowcast for waves 3 and 5 of 2013 with the naive approach, the model without search volume, and the model with search volume is 3.35, 3.27, and 2.38, respectively. For 2013 models with and without the internet search volume nowcast May-Jun and Sep-Oct harvest better than the naive approach. Indeed, the model with internet search volume nowcasts 27% better than the model without internet search volume and 29% better than the naive prediction.

The MAE of the nowcasts over the four periods considered in 2012 and 2013 is 1.88, 2.14, and 1.48, respectively, for the naive approach, the model without search volume, and the model with search volume. The model with internet search volume performs best, followed by the naive approach and then closely by the model without internet search volume. Overall, the model with internet search volume nowcasts 31% better than the model without internet search volume and 22% better than the naive prediction.

## Discussion

Fisheries in the United States are managed with hard caps on harvest for any given year. Staying under the cap throughout the year can be difficult, especially for recreational fishing where preliminary harvest estimates are usually unavailable until more than a month after the monitoring period is completed. This can be long after the season ends. We examined the potential for using nowcasting with internet search information to generate predictions of the recreational harvest before official estimates are available. The Gulf of Mexico recreational red snapper fishery has been managed for over ten years using effort-based controls such as bag limits and seasonal closures. During this time, the bag limit has been cut in half, and the season been reduced from nearly 200 days to less than 20 days. Despite these measures, harvest has exceeded the allowable catch in recent years, and the lag in reporting harvest estimates is a significant impediment to successfully managing the fishery.

Estimates of the recreational harvest of red snapper in the Gulf of Mexico are generated in bi-monthly waves and preliminary estimates are available around 45 days after the completion of each wave. Harvest estimates from the same wave in the previous year are a simple, intuitive proxy for the estimate of harvest in the wave just completed. This “naive” approach works well when fishery conditions (seasons, angler effort, and catch rates) are relatively stable. Indeed, the naive approach produced the best nowcasts for the open waves of 2012 when the season was similar to the previous year. However, we show that the naive approach can be problematic when current fishery conditions are considerably different than conditions during the previous year. For instance, a harvest prediction for the current period will not be available if the same period was closed to fishing during the previous year. In these cases, a more flexible modeling approach can be helpful.

Our results support a more flexible modeling approach for nowcasting harvest during 2013 when harvest levels were unprecedented due to changes in seasons, angler effort, and average fish size. A regression model using internet search information generates nowcasts that are nearly 30% better than the naive prediction and the the model without search information. This result is consistent with improvements in nowcasts with internet search information found in other (non-fishery) applications [6]. In general, the use of internet search information always improved the fit of our harvest regressions and increased the accuracy of the nowcasts. This suggests that there might be an underlying behavioral link between harvest levels and internet search volume (See Figs 2 and 3). More research is necessary to determine if correlation between harvest levels and internet search volume persists in other fisheries and different resource management situations. Researchers much be careful to separate behaviorally-relevant search terms from spuriously correlated terms [19, 20]. Survey or interview research might also help understand the nature of the behavioral link, if any, between internet search and harvest.

The regression modelling approach we propose is also useful because it makes uncertainty explicit. In the face of missing or delayed information on current harvest, fishery managers need to decide on the amount of overharvesting risk they are willing to tolerate. We show how our approach can be used to quantify this risk in terms of a probability of exceeding the quota. Understanding the risk of exceeding the harvest quota is very important when managers consider whether there is enough quota remaining to reopen the season for some period in the later part of the year as was done in the red snapper fishery during 2013. Due to data reporting delays in 2013, managers were not aware that the quota had already been exceeded by a considerable margin during the summer season. Our nowcasting model with internet search information would have predicted a nearly 20% probability that the quota had been exceeded during the summer season of 2013. Fishery managers could have considered this information when deciding to reopen the fishery in the fall of 2013. Staying within quotas has become more important recently for U.S. recreational fisheries where accountability measures have been introduced that require overages in one year to be “repaid” in the following year with tighter regulations on harvest.

It is important to note that the relationship between the “red snapper season” search term and red snapper harvest might change over time. This could occur if, for example, there are changes in the number of people using Google to find information about fishing relative to the population of potential anglers in the fishery of interest. In our case study, we attempted to keep the forecast model current by re-estimating the model using all of the data available up to the point of the nowcast. In other cases, though, if the relationship between internet search activity and harvest is suspected to have changed significantly, then it may be necessary to drop some of the data from early years when re-estimating the model.

There are several ways the nowcasting approach presented in this paper could be extended. First, as already noted, it would be useful to see if the harvest nowcasting improvements we found with internet search volume hold for other fisheries or other natural resource management situations. Second, the regression model used to predict harvest could include the internet search volume measured at different times during each two-month period. Google Trends provides weekly estimates of search volume which gives eight potential estimates that could be used in our model of bi-monthly harvest. Lastly, the regression approach could potentially be improved with other timely indicators of harvest behavior. This could include the volume of other internet search terms or multiple search terms combined in an index of sportfishing interest. In addition, the early work using social media activity to nowcast economic phenomena is promising [21]. There has been some work in fisheries using a small scale social network to understand the distribution of fishing effort [22]. It is worth exploring whether information on social media volume for terms related to recreational fishing regulations and activity can be used to improve models used to nowcast harvest activity. As a final note, we caution that nowcasting cannot replace traditional data collection methods in providing scientifically valid estimates of harvest. Nowcasting can provide useful information in some cases until existing data collection methods are able to provide more timely harvest estimates.
